# The Ætiology of Malignant Disease

**Published:** 1909-02-06

**Authors:** R. T. Hewlett

**Affiliations:** Professor of General Pathology and Bacteriology, King's College, London


### Hospital Clinics.

### THE ÆTIOLOGY OF MALIGNANT DISEASE.

By R. T. HEWLETT, M.D., F.R.C.P.; Professor of General Pathology and Bacteriology, King's College, London.

(A Lecture delivered at the London School of Clinical Medicine).

### The Universality of Cancer.

It may be said with regard to malignant disease—which I shall use as synonymous with cancer for the purpose of this lecture, reserving the term carcinoma for growths of epithelial type—that it is met with in all classes of vertebrates. Thus among mammalia it is found especially in the dog, and quite commonly in the ox, horse, cat, mouse, rat, and others. It is not common in birds, though several typical cases have been recorded. Malignant disease has not been reported in reptiles, but it has been found in amphibians and fish.

Cancer is not a disease of civilised nations only; it is met with by no means rarely among savages, and in certain cases it becomes very frequent in particular localities. Thus in the Cancer Research Fund report are figured cases of scirrhous carcinoma from Khartum, epithelioma of the jaw from Fashoda, sarcoma from Basutoland and from New Guinea, and so on.

It is perhaps true that cancer is relatively infrequent among uncivilised peoples, but it must be remembered that savages do not live to the cancer age nearly so often as do individuals of the civilised white races; probably the percentage of those who reach the age of forty among Europeans is twice as great as among native tribes.

### The Parasitic Hypotheses.

Among the hypotheses of the ætiology of malignant disease is that which assigns the cause to a microparasite. At one time a good deal of stress was laid upon the resemblances between malignant disease and the infective granulomata, especially tuberculosis. Although at first sight there is a certain amount of analogy, it is in reality superficial. If you inject a microparasite—*e.g.* a tubercle bacillus—the primary lesion is derived from the cells of the host, and so also are the secondary growths due to spread of the infection. This is quite different from what happens in the case of malignant disease. Here the secondary growths, save for a certain amount of inflammatory reaction, are not formed from the cells of the part affected, but are derived by growth of pieces separated directly from the primary tumour.

Moreover, with regard to this parasitic hypothesis, search has been made for bacterial forms, but quite without success. At one time certain yeast-like forms were described as the cause of malignant disease. With these various observers were able to produce appearances not unlike those of cancer; but all are now agreed that these tumours were really infective granulomata, and not true malignant disease.

Then the protozoal hypotheses arose. Notable among these were the speculations based upon the discovery of Ruffer's and Plimmer's bodies, found in carcinoma cells as round or ovoid structures with highly refractile capsules, and with occasionally appearances of nuclei. This hypothesis also has almost died out, and the supposed protozoal parasites are now regarded as cell inclusions of leucocytes or other cells which have been ingested, or as being colloid and other forms of degeneration.

Recently the bacterial hypothesis was revived by Doyen, who described the *Micrococcus neoformans*. But all recent work shows that this organism is not in any way specific; it is an ally of the ordinary staphylococcus.

During the last four or five years the parasitic hypothesis has been losing ground, though it cannot be said to be disproved altogether. If there is a parasite which is the cause of cancer, it must, I think, be an intracellular one.

### Cancer Houses and Personal Infection.

There is one other point to be noted on the question of the infectivity of malignant growths. It is sometimes stated that cancer is infectious, and cancer houses are described. No doubt there is apparently an abnormal frequency of cancer in certain houses, but the cases are so few that they may be merely due to coincidence; and it has been shown mathematically and statistically that there is no proof that special houses infected with cancer exist. At the same time it is true that certain districts are more affected than others, particularly valleys, water-logged localities, and those with cold, clay soil.

As regards infection from person to person, it has been found from experiments on animals, especially mice, that cancer can be implanted from diseased into healthy animals, and that nearly 100 per cent. of successes may follow such inoculations. Such growths are, however, extremely specific: that is, a mouse cancer cannot be implanted successfully into a rat; and it may even be impossible to transfer the tumours from the mice of Berlin to those of London. Still, it must be acknowledged that there is a possibility of the infection of a healthy person from a diseased one, though this must be very unlikely to occur.

There is little doubt that cancer may be implanted from one adjacent surface on to another in the same patient, but it is only by direct contact that such implantation can occur. Moreover, a certain dose of cancer infection seems to be necessary in such cases of transference.

Yet another point, which bears on the false analogy between cancers and the infective granulomata, is the fact that an inoculation of this sort is a transplantation rather than an infection. None of the tissues of the inoculated animal takes part in the growth, the whole of which is derived solely from the implanted mass of cancer tissue.

### Non-Parasitic Hypotheses.

Of the other hypotheses advanced to account for the genesis of malignant disease, Cohnheim's must be mentioned. He propounded the doctrine that malignant growths arise from the abnormal activities of certain embryonic tissues. According to this hypothesis, during development certain groups of cells, instead of developing normally for their proper uses, lie dormant and form "embryonic rests." In consequence of some stimulus, such as some alteration of the blood supply, or irritation, they start into abnormal activity and form a malignant growth. This theory does seem to account for some varieties of malignant disease; for example, new growths of the kidney are, as we know, often formed from adrenal rests. But it is doubtful whether it will apply to the majority of cases.

Then there is Beard's hypothesis, in connection with which the treatment of malignant disease by means of ferments was invented. This observer believes that in development there is not only the one proper embryo, but also secondary and rudimentary embryos; and that these wander to different parts of the body and finally may waken into activity and start malignant growth. He compares the multiplication of the cells in a growth to the asexual generation, which alternates with the ordinary sexual process in certain animals.

Again, Ribbert believes that the development of a tumour is due to detachment of certain cells, which lose the influence of their proper stimuli and take an abnormal growth. It is difficult to accept this explanation, because one would imagine that displacement of cells must take place in every surgical operation and in every other form of trauma, and yet comparatively few malignant tumours develop.

### The Cytology of Cancer.

Four or five years ago Farmer, Moore, and Walker made some very interesting observations on the cytology of malignant disease. They critically examined the nuclear changes in the cells of malignant growths. In ordinary cell division certain changes take place in the nucleus previous to division, consisting in the formation of a spindle-like body on the equator of which the chromatin of the nucleus becomes arranged in V-shaped masses, or chromosomes. The number of these chromosomes for any one species of animal is constant in all the various tissues of the body.

In man the number of these chromosomes is thirty-two. Each chromosome splits longitudinally, and the halves of each then travel towards the two poles to form the new nuclei. Now at one period in the development of the embryo there is a reduction of the number of chromosomes in those cells from which the reproductive organs are being formed, and also these chromosomes divide transversely instead of longitudinally.

Now in examining cancer, Farmer, Moore, and Walker came to the conclusion that this heterotype form of mitosis occurs in cancer cells, which thus resemble embryonic reproductive tissue more than ordinary body tissue. They applied the term gametoid to this heterotype cancer tissue to signify the kinship with gametogenic or reproductive tissue. These researches suggested to many minds that in some way there is in cancer a change towards a reproductive type of cell. The cytological investigations along these lines have been continued; but Bashford and Murray do not accept the conclusions of Farmer, Moore, and Walker, though they admit the irregularity of cancer mitoses.

### A Suggestion.

What view, then, can be taken of the ætiology of malignant disease? It seems to me that it is unnecessary to invoke the aid of parasites or of embryonic remnants for the explanation of cancer. You have, of course, the one remarkable fact, which to my mind has not received the attention it should do, that during growth something controls that growth and the amount of new tissue formed. There must be some delicate mechanism which controls cell growth, and I feel convinced myself that there is something that does this, though I do not know what it is. If you weaken that mechanism, I cannot see why cells should not go on growing and multiplying so long as they have sufficient nutriment.

The influence of irritation is also to be considered. One example of this influence is the malignant changes which have followed $x$-ray dermatitis. There is, again, the fact that chemical agents will produce cancer, which is unduly prevalent among certain workers in petroleum and various anilines. Physical injury will also produce malignant disease; the familiar instance of the clay pipe shows this, but it is even better illustrated by the development of cancer of the anterior abdominal walls in many of the inhabitants of Thibet. These people, who thus get cancer in such an unusual situation, carry tied to the front of their bellies a small charcoal fire in a basket to keep themselves warm, and are in consequence frequently scorched and burnt. Then, again, Jews and Mohammedans hardly ever suffer from cancer of the penis, because they all undergo circumcision at an early age. Cancer of the mouth, too, is unduly common in India, owing to the habit of chewing betel nut.

Whether these stimuli of irritation act on embryonic residues, or whether they weaken the normal restraining influence which prevents the undue multiplication of cells, at any rate it is well established that an external stimulus may result in cell proliferation, as seen in the horniness of the skin which develops in certain situations. This is all that can be said for certain about the ætiology of malignant disease.

We generally hold that carcinoma is developed from either epiblast or hypoblast, and sarcoma from mesoblast. The trend of opinion now is that certain cases of apparent deviation from this rule are due to transformation of the stroma of the tumour, derived from the host. That is to say, that when a carcinoma seems to be transformed into a sarcoma the transformation is not of the cells of one growth into those of the other, but is merely the over-development of the stroma as compared with that of the epithelial cells of the original growth.

A Forecast for the Future.

There are one or two points about the direction in which progress may be expected in the future. We know, of course, that human beings are sometimes, though very rarely, cured of cancer spontaneously. It is also found that if a mouse is inoculated with malignant disease and escapes the development of a tumour, it is then generally immune to further inoculation. Moreover, it has been proved that the previous injection of a little mouse blood from another animal seems to prevent the development of an inoculated carcinoma.

Then, again, we know that mice injected with an emulsion made from mouse embryos are similarly to a large extent immunised; and still more are they protected if normal tissue of a similar type to that wherein the carcinoma has been growing is injected. These experiments certainly suggest that it may be possible by the injection of cellular products to procure immunity to cancer, though this does not necessarily include the cure of cancers already formed.

The great difficulty lies in the specificity of cancer; that is in the differences between the cancer cells of one species of animal and another. To make any experiments, therefore, along these lines with human beings, human cellular products would probably be necessary. The problem of cancer is by no means as yet solved, but it does seem as if we are getting a little way along the road.

